# Clinical Effectiveness of a Combination of Black Elder Berries, Violet Herb, and Calendula Flowers in Chronic Obstructive Pulmonary Disease: The Results of a Double-Blinded Placebo-Controlled Study

**DOI:** 10.3390/biology9040083

**Published:** 2020-04-22

**Authors:** Tatiana V. Kirichenko, Igor A. Sobenin, Yuliya V. Markina, Elena V. Gerasimova, Andrey V. Grechko, Dmitry A. Kashirskikh, Elena B. Romanenko, Wei-Kai Wu, Alexander N. Orekhov

**Affiliations:** 1 Laboratory of Infection Pathology and Molecular Microecology, Institute of Human Morphology, 117418 Moscow, Russia; t-gorchakova@mail.ru (T.V.K.); igor.sobenin@gmail.com (I.A.S.); a.h.opexob@gmail.com (A.N.O.); 2 Laboratory of Medical Genetics, National Medical Research Center of Cardiology, 121552 Moscow, Russia; 3 Laboratory of Angiopathology, Institute of General Pathology and Pathophysiology, 125315 Moscow, Russia; dim.kashirsckih@gmail.com; 4 Department of Systemic Rheumatic Diseases, V.A. Nasonova Institute of Rheumatology, 115522 Moscow, Russia; gerasimovaev@list.ru; 5 Federal Research and Clinical Center of Intensive Care Medicine and Rehabilitology, 109240 Moscow, Russia; avg-2007@yandex.ru; 6 Department of Molecular Basis of Ontogenesis, Belozersky Institute of Physical and Chemical Biology, Moscow State University, 119234 Moscow, Russia; romanenkoeb@mail.ru; 7 Department of Internal Medicine, National Taiwan University Hospital, Bei-Hu Branch, Taipei 108, Taiwan; weikaiwu0115@gmail.com

**Keywords:** COPD, anticytokine therapy, natural preparation, Inflaminat

## Abstract

Chronic obstructive pulmonary disease (COPD) is a multifactorial disease, in which systemic inflammation plays a key role. This 6-month randomized double-blinded placebo-controlled study evaluates the possible effect of natural preparation Inflaminat on clinical symptoms of COPD, indicators of respiratory function, and exacerbation frequency in 60 patients with moderate severity of COPD. Inflaminat is a combination of natural ingredients black elder (*Sambucus nigra* L.) berries, violet (*Viola tricolor* L.) herb, and calendula (*Calendula officinalis* L.) flowers. The preparation has been previously demonstrated to possess anticytokine and anti-inflammatory effects in experimental studies. In present study, COPD dynamics were evaluated by means of BCSS (Breathlessness, Cough, and Sputum Scale) and spirometry tests. It was shown that 6-months Inflaminat administration led to significant decrease of BCSS points from 3.0 ± 0.6 to 1.9 ± 0.7, (*p* = 0.002) as well as significant increase of FEV1 from 66 ± 18% to 73 ± 17%, (*p* = 0.042); there were no beneficial dynamics in placebo group. Side effects associated with preparation administration were not identified. The results of the study suggest that Inflaminat may be employed in treatment of patients with moderate severity of COPD, since it has a positive effect on COPD symptoms according BCSS and indicators of respiratory function FEV1.

## 1. Introduction

Chronic obstructive pulmonary disease (COPD) is a multifactorial disease, in which systemic inflammation plays a key role. The inflammatory response in COPD is determined by the activation of epithelial cell and macrophage dysfunction in the respiratory tract [1]. Multiple cytokines are implicated in chronic inflammation associated with COPD. Proinflammatory cytokines, such as TNF-α and IL-1β, amplify the inflammatory response [2,3]. However, the role of individual inflammatory mediators in the pathogenesis of COPD is difficult to assess, since each mediator has pleiotropic effects in the development of the pathology. In particular, increased concentrations of TNF-α and IL-6 were demonstrated in induced sputum in stable COPD [4,5]. Immunoreactive cells positive for interleukin-1α (IL-1α), IL-4, IL-6, IL-7, IL-8, IL-10, IL-12, and TNF-α were detected in lung tissue of COPD patient significantly higher than in control group [6]. Recent studies have demonstrated that polymorphisms in proinflammatory genes are significantly associated with the susceptibility and severity of COPD [7,8].

In recent years, the natural anticytokine preparation Inflaminat, a combination of calendula, elderberry, and violet, was developed and characterized. The preparation was shown to possess a pronounced anti-inflammatory effect in vitro, ex vivo, and in vivo, and was tested in a pilot clinical study in patients with reactive arthritis [9,10]. The results of the ex vivo study demonstrated that Inflaminat suppressed the proinflammatory activity of human serum by 22–38%, namely, Inflaminat administration led to significant suppression of the expression of inflammatory cytokines TNF-α and IL-1β in primary culture of human macrophages induced by serum taken after Inflaminat administration and serum from control patients not receiving any preparations [9]. A study conducted on an animal model of aseptic inflammation in connective tissue of rats’ skin, induced by cryodamage, showed that treatment with Inflaminat resulted in a significant reduction of the recovery time for the number of hematogenous cells and mast cells in comparison with control group without any preparations [9]. In a pilot clinical study in patients with reactive arthritis, Inflaminat administration was associated with positive clinical dynamics manifested in a decrease of the number of affected joints and pain intensity. Inflaminat was shown to possess a clinically relevant anti-inflammatory effect comparable to that of nonsteroid anti-inflammatory preparations. A tendency to reduce the plasma level of TNF-α in patients with reactive arthritis was observed, but the results did not reach statistical significance due to a small sample size and short period of follow-up [9]. This study aims to evaluate Inflaminat efficacy for treatment of chronic inflammatory processes and possible effects of Inflaminat on the clinical symptoms of COPD, indicators of respiratory function, and exacerbation frequency of COPD patients.

## 2. Materials and Methods

### 2.1. Subjects

This randomized double-blinded placebo-controlled study was performed in accordance with the Declaration of Helsinki. The present study is the analysis of subgroup from the randomized double-blinded placebo-controlled clinical study of the effect of Inflaminat on carotid atherosclerosis progression (NCT01743404). The study protocol was approved by the Institute for Atherosclerosis Research Committee on Human Research of 107-15. The substudy measuring the effect on COPD as well as the substudy of the efficacy of Inflaminat in patients with reactive arthritis was prespecified in the trial protocol to evaluate anti-inflammatory effectiveness. All study participants signed informed consent and fulfilled the following inclusion criteria: male gender, age 40–70 years old, smoking more than 10 pack/years, indicators of respiratory function—forced expiratory volume in 1 s (FEV1) up to 80–50% from normal, forced expiratory volume/forced vital capacity (FEV1/FVC) ratio less than 70%, ∆FEV1 less than 12%, and 200 mL in the pharmacological test with Salbutamol. Exclusion criteria were regular intake of inhaled or systemic steroids and continuous intake of other anti-inflammatory medications.

### 2.2. Study Design

Subjects were randomly divided in two equal groups to receive Inflaminat or placebo in the same regimen during 6 months. The study included three visits: at baseline, after 3 months and final visit after 6 months of follow-up. The endpoints of the study were severity of clinical symptoms, frequency of exacerbations and number of hospitalizations, and the results of spirometry tests—FEV1 and FEV1/FVC. Clinical symptoms were evaluated according BCSS (Breathlessness, Cough, and Sputum Scale) [11], a three-item questionnaire that evaluates the severity of COPD symptoms by 5-point Likert scale to (0 = no symptoms, 4 = severe symptoms). The course of disease (frequency of exacerbations and number of hospitalizations, per month) and BCSS were assessed within one year prior to inclusion in the study according to anamnesis and twice during the follow-up period.

### 2.3. Production and Dispensing of Preparations

Natural anticytokine preparation used in this study was officially registered as a dietary supplement “Inflaminat” and manufactured by INAT-Farma (Moscow, Russia). Inflaminat was presented as 500 mg capsule that contain: 165 mg of black elder berries (*Sambucus nigra* L.), 165 mg of violet tricolor herb (*Viola tricolor* L.) and 165 mg of calendula flowers (*Calendula officinalis* L.). The amount of active materials contained in Inflaminat capsules maintained constant. Placebo capsules had the same capsule size and color and contained excipient only. Study participants were instructed to take three capsules daily for 6 months.

### 2.4. Statistical Methods

Results were expressed in terms of means and standard deviations. Significance of differences was evaluated using SPSS 12.0 statistical program package (SPSS Inc., Chicago, MI, USA). Significance was defined at the 0.05 level of confidence. Changes from baseline to the mean of follow-up visits were analyzed by a two-way ANOVA and paired two-tailed t-Test. The datasets used to support the findings of this study are available from the corresponding author upon request.

## 3. Results

**Figure 1:**
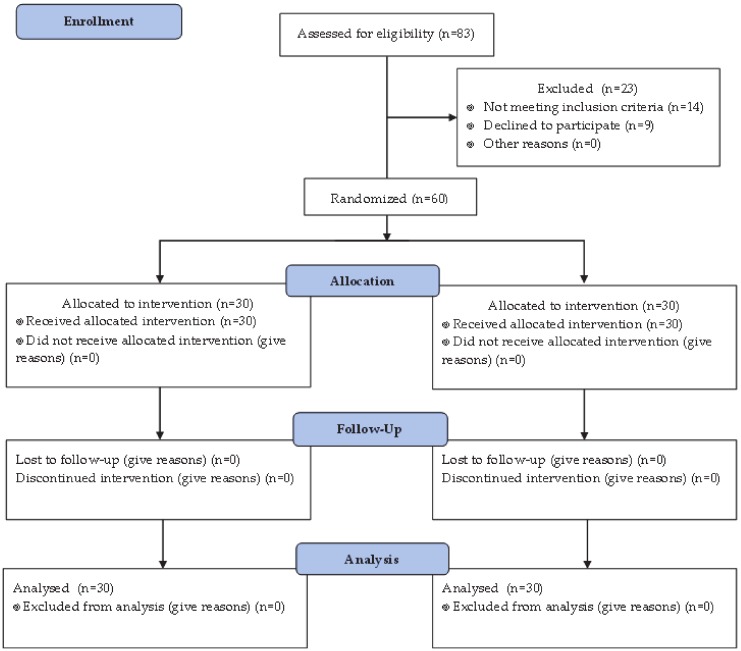
Consort flow diagram of the study on the effect of natural anti-cytokine preparation Inflaminat on COPD dynamics.

A total of 60 male patients were included in the study, 30 in the Inflaminat group and 30 in the control group. All study participants underwent three planned visits at baseline, and after 3 and 6 months of the follow-up, no patients were excluded from the study. Consort flow diagram for the study is presented at Figure 1. Side effects associated with preparation administration were not identified. According to the inclusion criteria, all study participants were smokers, aged 42–67 years, had II stage of COPD (moderate severity) and did not receive anti-inflammatory preparations regularly.

Baseline characteristics of the study participants are presented in Table 1. There was no statistically significant difference between groups in age, BCSS points, frequency of exacerbations during one year before the study, and indicators of respiratory function.

Dynamics of base clinical characteristics are presented in Table 2. There were no significant changes of clinical parameters in both groups during first three months of follow-up. During 6 months of follow-up, BCSS points decreased significantly in the Inflaminat group from 3.0 ± 0.6 at baseline to 1.9 ± 0.7 on last visit (*p* = 0.002) and number of exacerbations per month decreased from 0.15 ± 0.09 at baseline to 0.12 ± 0.11 on the last visit, but this reduction did not reach statistical significance (*p* = 0.057). In placebo group, there were not statistically significant changes in COPD dynamics during follow-up period.

After 6 months, BCSS points in Inflaminat group decreased due to significant reduction of cough and sputum production (Table 3). At the same time, there were no significant dynamics of clinical parameters in the control group up to 6 months of observation period. Regarding the frequency of exacerbations that required hospitalization, no significant changes were found either in Inflaminat or control group.

There was a significant increase of FEV1 in the Inflaminat group from 66 ± 18% to 73 ± 17%, (*p* = 0.042) (Table 4). The ratio of FEV1 to FVC increased from 61 ± 13% at baseline to 66 ± 18 on last visit after 6 months of follow-up, but the change was not significant (*p* = 0.076). In the control group, no significant changes of these indicators were observed.

## 4. Discussion

The results of this study demonstrate that natural preparation Inflaminat had a positive effect on COPD parameters in patients with moderate severity of disease during long-term administration. After 6 months of Inflaminat use, cough and sputum production were decreased. As for indicators of respiratory function, Inflaminat use leads to increase of FEV1. The beneficial effect on frequency of COPD exacerbations and elevation of the ratio of FEV1 to FVC was also observed, but these changes did not reach significance. The positive effect of Inflaminat on the dynamics of COPD can be explained by the anticytokine mechanism of action of the preparation previously demonstrated in in vitro and ex vivo studies. It was shown that a single dose of Inflaminat intake led to significant suppression of the blood serum-induced expression of proinflammatory cytokines such as IL-1, TNF-α, HLA-DR, and ICAM-1, in primary culture of monocytes/macrophages [9].

Currently, anticytokine therapy is regarded as a promising therapeutic approach for treatment of COPD. The efficacy of direct anticytokine medications, as well as multieffect preparations possessing anticytokine potential, is being actively studied [12,13,14,15,16,17,18,19,20,21,22,23]. Statins, HMG-CoA inhibitors, are widely known as preparations that have multiple effects, including anticytokine and anti-inflammatory potential [13,14]. Statin therapy could be effectively used in COPD patients with increased cardiovascular risk. COPD patients receiving statins have a lower frequency of COPD exacerbations, hospitalizations, and antibiotic prescriptions than patients not receiving statins [15]. Significantly decreased concentrations of IL-1β, IL-2, IL-4, IL-8, IL-10, IL-12p70, and TNF-α were found in COPD patients receiving statin therapy in comparison with COPD patients not receiving statin therapy (*p* < 0.05) [16]. It has been demonstrated that metformin and pioglitazone have pleiotropic effects and can be effective for COPD treatment in patients with type 2 diabetes, since they also have anticytokine action [17]. Monoclonal antibodies have been evaluated for treatment of COPD, but they are directed towards specific cytokines, while there is no dominant role for any single cytokine in the inflammatory response observed in COPD. This may explain the lack of sufficient effect of these therapies in COPD patients [18]. In particular, TNF blockers failed in COPD treatment because the inflammatory response was mediated by other inflammatory cytokines [19]. Besides, such therapies may have severe adverse effects. Nevertheless, cytokine blockade is a novel treatment strategy, and several monoclonal antibodies are being evaluated for COPD treatment, including dupilumab (anti-IL4), reslizumab and benralizumab (anti-IL5), and lebrikizumab and tralokinumab (anti-IL13) [20]. Natural preparations are often used as therapeutic agents in combination with pharmacotherapy or as monotherapy for COPD treatment. Mechanisms of anti-inflammatory action of herbal preparations in COPD are being actively studied. A herbal preparation HemoHIM reduced the inflammatory cell count and levels of TNF-α, IL-6, and IL-1β, in the broncho-alveolar lavage fluid [24]. Green tea consumption was associated with positive dynamics of expiratory function parameters [25]. Chinese Herbal Medicine improves clinical symptoms of COPD in several studies as add-on therapy [26]. It was also shown that two Chinese herbal formulas decreased inflammatory cytokines IL-8, and IL-17, IL-1β, IL-6, and TNF-α [27].

Anticytokine activity is one of the possible mechanisms of Inflaminat’s activity for COPD, since natural products are usually characterized by pleiotropic effects in treatment of chronic inflammation. According Dr. Duke’s Phytochemical and Ethnobotanical Database, the numerous active materials contained in Inflaminat have the potential to decrease clinical symptoms of COPD. In particular, black elder berries contain beta-carotene and magnesium, which possess antiasthmatic properties; citric acid, which has antibacterial and antiseptic effects; and ascorbic acid, which has multiple benefitial properties for patients with COPD such as mucolytic, antiasthmatic, anti-inflammatory, antibacterial, and antiseptic. Violet herb contains methyl-salicylate that possesses anti-inflammatory and antiseptic effects, tannin, and p-coumaric acid with antibacterial activity. Calendula flowers contain numerous active compounds that possess anti-inflammatory activitiy: ascorbic acid, caryophyllene, chlorogenic acid, lupeol, oleanolic acid, stigmasterol, vanillic acid, and rutin [28].The main limitations of this study are short observation period and small sample size. Correspondingly, some of the obtained results, such as exacerbation frequency and ratio FEV1/FVC, did not reach statistical significance. Another important limitation is the fact that laboratory tests of inflammation were not performed; only clinical dynamics and spirometry were evaluated. As a rule, natural preparations have pleotropic effects in the human body, and determining the level of cytokines, in particular, would allow one to obtain a clearer understanding of the mechanisms of Inflaminat positive action in COPD. However, present data could be useful for sample size, and the statistical power calculation of a new study and significant results are the base for the evaluation of inflammatory parameters in the new study design.

## 5. Conclusions

Our results suggest that natural preparation Inflaminat may be employed in patients with mild or moderate severity of COPD, since it demonstrated a positive effect on clinical symptoms of disease and indicators of pulmonary function. Since the anticytokine effect of Inflaminat was demonstrated in recent laboratory experiments and its positive clinical effect as well as an absence of adverse effects was shown in other clinical studies, we suggest that the potential use of natural anticytokine therapy with Inflaminat may have beneficial effects during long-term administration in diseases associated with chronic inflammation, in particular, COPD.

## Figures and Tables

**Table 1:** Baseline characteristics and pulmonary function of study participants.

Indicators	Inflaminat	Placebo	*p*
Age, years	54 ± 4	55 ± 3	0.785
Number of exacerbations, per month	0.15 ± 0.09	0.18 ± 0.10	0.163
BCSS, points	3.0 ± 0.6	3.0 ± 0.5	0.874
FEV1/FVC, %	66 ± 17	68 ± 25	0.608
FEV1, %	61 ± 12	62 ± 15	0.695
∆FEV1, %	5 ± 4	6 ± 3	0.702

**Table 2:** Course of disease during follow-up period.

Indicators	Group	12 Month Before Inclusion	0–3 Months of Follow-Up	3–6 Months of Follow-Up
BCSS, points	Inflaminat	3.0 ± 0.6	2.8 ± 0.6	1.9 ± 0.7 *
	Placebo	3.0 ± 0.5	2.8 ± 0.7	2.9 ± 0.6
Number of exacerbations, per month	Inflaminat	0.15 ± 0.09	0.15 ± 0.08	0.12 ± 0.11
	Placebo	0.18 ± 0.10	0.17 ± 0.10	0.19 ± 0.13
Hospitalizations due to COPD exacerbation, per month	Inflaminat	0.07 ± 0.04	0.06 ± 0.07	0.07 ± 0.05
	Placebo	0.08 ± 0.06	0.07 ± 0.04	0.08 ± 0.07

*—ANOVA, *p* < 0.05.

**Table 3:** Dynamics of clinical symptoms.

Symptoms	Group	Baseline	3 Months	6 Months
Cough, points	Inflaminat	2.9 ± 0.7	2.6 ± 0.5	1.2 ± 0.7 *
	Placebo	3.1 ± 0.6	2.9 ± 0.6	3.0 ± 0.7
Breathlessness, points	Inflaminat	2.8 ± 0.5	2.9 ± 0.5	2.6 ± 0.6
	Placebo	2.9 ± 0.5	2.7 ± 0.7	2.8 ± 0.6
Sputum production, points	Inflaminat	3.2 ± 0.5	2.8 ± 0.7	1.8 ± 0.7 *
	Placebo	2.9 ± 0.5	2.8 ± 0.6	2.9 ± 0.6

*—ANOVA, *p* < 0.05.

**Table 4:** Pulmonary function dynamics.

Indicators	Group	Baseline	3 Months	6 Months
FEV1/FVC, %	Inflaminat	61 ± 13	63 ± 16	66 ± 18
	Placebo	62 ± 15	62 ± 12	64 ± 15
FEV1, %	Inflaminat	66 ± 18	68 ± 16	73 ± 17 *
	Placebo	68 ± 25	69 ± 22	71 ± 21

*—ANOVA, *p* < 0.05.
